# The League of Mercy

**Published:** 1909-12-25

**Authors:** 


### THE LEAGUE OF MERCY.

### TENTH ANNUAL MEETING OF PRESIDENTS.

### A Successful Year.

The tenth annual meeting of Presidents of the League of Mercy was held in the Board Room of Westminster Hospital on Monday, December 20th, the Right Hon. the Lord Farquhar, G.C.V.O., Chairman, presiding. The minutes of the last meeting were read and duly confirmed.

### Letter from the Prince of Wales.

Mr. E. W. Wallington (Hon. Registrar of the Order of Mercy) read the following letter from his Royal Highness the Prince of Wales (Grand President) :—" Their Royal Highnesses wished to say how pleased they are to hear that the number of districts in which the organisation of the League of Mercy is at work is still increasing, and that some sixty new Vice-Presidents have been enrolled during the year. It is very gratifying to their Royal Highnesses to find that over and above the large sum handed over to the King's Fund, the amount distributed by the League among provincial hospitals had been augmented by £500, making the contribution to those institutions for 1909 nearly £2,000. Another satisfactory feature of the past year is the increase of small subscribers among the poorer citizens of London. And the Grand President and Lady Grand President are interested to learn that special collecting cards have been issued to meet the case of the penny subscribers. The Prince and Princess of Wales desire to renew their sincerest thanks to the President and Lady President for giving so much of their time to the services of the League, and to congratulate them, and all associated with them, on the very successful results of their work.

### The Chairman's Speech.

Lord Farquhar said it was a very pleasant duty to thank their Royal Highnesses for their address. He thought all would agree with him that most of the success of the League of Mercy was due to the unfailing support and interest which the Prince and Princess of Wales had always taken in its welfare. They never missed an opportunity of doing all they could to strengthen and increase its work and prosperity; and each year they gave a garden party so that they might have the opportunity of meeting personally the members of the League who could attend. Nine hundred people attended the party this year at Marlborough House. He had also to thank H.R.H. the Duchess of Albany, H.R.H. Princess Alexander of Teck, H.H. Princess Victoria of Schleswig-Holstein, for the great support which the League had received from them, and always had received in the past. Their Royal Highnesses and their Highnesses had been present at endless meetings, and had taken during the year an immense interest in everything connected with the League. And though he had to regret that there had been some resignations during the year, there had been an increase of fifty-seven vice-presidents. He regretted the death of several zealous workers, and everyone would agree in deeply deploring their loss, and that they had always done their best for the welfare of the League. Though Lady Dimsdale had been ill, she was now better, and she had herself arranged the meeting at the Mansion House, and in the City she had thoroughly organised the work. She had always been most active on behalf of the League. He also mentioned with gratitude the name of Mr. Cremien Javal, who, up to the time Lady Dimsdale took over the work in the City of London, acted as one of the vice-presidents and looked after the League in that district. He had collected during the last ten years nearly £2,000. In the present year, though it had not been a very good one for collecting money for charities or any other purpose—he did not know why—(laughter)—they were still able to hand over £19,000 to the Hospital Fund. (Applause.) That was outside the sum given to the country hospitals. That was satisfactory, as there had been no concerts or demonstrations to bring them in mind of the work. He hoped that next year something fresh would be done for the special work of the League. He was very anxious that all the workers should have encouragement to go on doing more and more every year. Since its formation, the League had subscribed £135,000 to the Hospital Fund, in addition to the grants to the county hospitals. He could only join humbly in the wish expressed by his Royal Highness, the President, that all the workers of the League might go on even more prosperously next year than heretofore, and that all would do their utmost for the benefit of the League.

### The Resolutions.

Mr. George Alexander proposed the following resolution : "That the Right Hon. the Lord Farquhar, G.C.V.O., be appointed Chairman; Mr. Frederick Green, J.P., Deputy-Chairman; and Colonel H. B. Hamilton, Hon. Secretary," respectively, and in doing so said that in those names was wrapped up the success of the League of Mercy, and he could not hope to better express the value of the services they had rendered than in the words the Chairman had used.

Mr. Edward Murray Ind, D.L., J.P., seconded the resolution. It had been well said that the success of the League was in the hands of the Chairman, Vice-Chairman, and Council to keep everything going, and the results of this year's efforts had shown how well their work had been done. He could bear testimony to the way in which the Albert Hall concert had stimulated further effort, and the question had often been asked when such another event would be held. The resolution was carried.

Mr. Alan H. Burgoyne proposed the following resolution : "That seven representative members of the Executive Committee be appointed : The Right Hon. the Lord Farquhar, G.C.V.O., Colonel H. B. Hamilton, Mr. Frederick Green, J.P., Mrs. R. L. Harrison, Mr. Edward Murray Ind, D.L., J.P., Lady Morrish, and Mr. Montagu Sharpe, D.L." Seconded by the Rev. W. H. Hornby Steer, M.A., and was carried.

### The Treasurer's Statement.

Sir Henry Burdett (the Hon. Treasurer) said there was one very encouraging fact in a year which had not been a good year for getting money—namely, that on the average contribution being taken of the 98 districts they were found to have produced £222 each, a very satisfactory average, though it was £1,000 short of the minimum aimed at. Although the League was constantly losing workers, it was also constantly gaining new ones, and the net profit upon the workers, as represented by the Vice-Presidents alone, during the year had been no less than 64. Until the League was founded, no one heard in this country of the principle of personal service. The League of Mercy was the mother of personal service applied as a doctrine to the work of charity in England. Imitation was said to be the sincerest form of flattery, and the League might well feel flattered that during the current year other organisations had come out and asked for workers to give a measure of personal service to other causes. Apart from the money raised, the League had exercised, as the Archbishop of Canterbury had testified, a great moral influence for good upon many thousands of people. Sir Henry then gave the returns of the results of the year's work in 98 districts.

Yield of each District in 1909.

South Kensington £1,522, as against £1,623, a decrease of £101.
St. George's, Hanover Square, £1,034, as against £974, an increase of £60.
Paddington (South) £896, as against £775, an increase of £121.
Fulham £864, as against £931, a decrease of £67.
Epsom—Esher and Warlingham £674, as against £581, an increase of £93.
Sevenoaks £521, as against £453, an increase of £68.
Marylebone East £519, as against £615, a decrease of £96.
Kingston—Surbiton (with Wandsworth and Putney) £518, as against £504, an increase of £14.
Westminster £514, as against £318, an increase of £196.
Strand £429, as against £412, an increase of £17.
Lewisham £367, as against £234, an increase of £133.
East Sussex £350, as against £383, a decrease of £33.
Hornsey and Finchley £336, as against £317, an increase of £19.
Kensington (North) £311, as against £394, a decrease of £83.
Ealing £299, as against £189, an increase of £110.
Tonbridge £296, as against £281, an increase of £15.
Mid-Essex £293, as against £267, an increase of £26.
Chertsey and Wokingham £269, as against £270, a decrease of £1.
Deptford—Brockley £267, as against £228, an increase of £39.
Guildford £257, as against £255, an increase of £2.
Hampstead £257, as against £327, a decrease of £70.
N. E. Sussex £241, as against £265, a decrease of £24.
Chelsea (decrease being due to workers having left the district) £230, as against £467, a decrease of £237.
Dulwich and Norwood £225, as against £212, an increase of £13.
Marylebone (West) £212, as against £659, a decrease of £447.
St. Pancras (South) £210, as against £1, an increase of £209.
Luton £209, as against £198, an increase of £11.
Eastbourne £203, as against £210, a decrease of £7.
City of London (Lady Dimsdale began to work it in October) £202, as against £123, an increase of £79.
Epping £197, as against £191, an increase of £6.
Uxbridge £195, as against £206, a decrease of £11.
Eye £193, as against £222, a decrease of £29.
Hertford £192, as against £236, a decrease of £44.
Lambeth—Brixton £181, as against £163, an increase of £18.
Richmond £173, as against £213, a decrease of £40.
St. Pancras (West) £168, as against £198, a decrease of £30.
Woolwich £167, as against £188, a decrease of £21.
Horsham £166, as against £158, an increase of £8.
Battersea £162, as against £118, an increase of £44.
S.E. Essex £158, as against £152, an increase of £6.
Hitchin £146, as against £104, an increase of £42.
Medway £139, as against £124, an increase of £15.
Maldon £138, as against £157, a decrease of £19.
Southwark (Bermondsey) £135, as against £112, an increase of £23.
Croydon £135, as against £91, an increase of £44.
Hackney, N. £135, as against £141, a decrease of £6.
Romford and Ilford £133, as against £129, an increase of £4.
E. Grinstead and Hayward's Heath £129, as against £166, a decrease of £37.
Reigate £121, as against £137, a decrease of £16.
Greenwich £114, as against £120, a decrease of £6.
Gravesend £107, as against £92, an increase of £15.
Enfield £100, as against £96, an increase of £4.
Ashford £95, as against £196, a decrease of £101.
Watford £89, as against £64, an increase of £25.
Lewes £82, as against £76, an increase of £6.
Tower Hamlets (Mile End) £81, as against £129, a decrease of £48.
Isle of Thanet £81, as against £96, a decrease of £15.
Islington (West) £74, as against £83, a decrease of £9.
Brentford £71, as against £297, a decrease of £226.
Hammersmith £71, as against £248, a decrease of £177.
Hythe £71, as against £80, a decrease of £9.
Streatham £67, as against £78, a decrease of £11.
Biggleswade £63, as against £102, a decrease of £39.
Limehouse £61, as against £71, a decrease of £10.
Tower Hamlets (Whitechapel) £60, as against £83, a decrease of £23.
Camberwell (North) and Newington (West) £57, as against £48, an increase of £9.
Harwich £57, as against £50, an increase of £7.
Wimbledon £51, as against £72, a decrease of £21.
Worthing £47, as against £54, a decrease of £7.
Lowestoft £45, as against £73, a decrease of £28.
Lambeth (Kennington) £43, as against £41, an increase of £2.
Rochester £43, as against £44, a decrease of £1.
Sudbury £43, as against £47, a decrease of £4.
Finsbury (East) £41, as against £42, a decrease of £1.
St. Albans £40, as against £55, a decrease of £15.
Faversham £39, as against £39, no change.
Bethnal Green £38, as against £31, an increase of £7.
St. Pancras (North) £38, as against £402, a decrease of £364.
Holborn £32, as against £55, a decrease of £23.
St. Pancras (East) £32, as against £25, an increase of £7.
Camberwell (Peckham) £29, as against £29, no change.
Walthamstow £28, as against £30, a decrease of £2.
Clapham £27, as against £26, an increase of £1.
Dartford £25, as against £20, an increase of £5.
Southwark (West) £24, as against £23, an increase of £1.
Saffron Walden £20, as against £25, a decrease of £5.
Berkshire £17, as against £727, a decrease of £710.
Paddington (North), district extinct £15, as against £227, a decrease of £212.
Colchester £11, as against £64, a decrease of £53.
Willesden £2.

Sir Henry Burdett stated the total result was that collections had been made in 98 districts, against 93 in the previous year, but the money paid in up to date was £17,783 from 90 districts, as against £17,894. In 50 divisions there had been a falling off amounting to £1,914; and in 44 an increase of £1,543, showing a net decrease of £371. From two districts no collections had yet been received or promised, though they were being worked, namely, Rotherhithe and Chichester. The total collections for the year, as estimated, amounted to £21,356, as against the total actually received last year £22,409, or a decrease of £1,053. But in the 1908 collections were included £579 from the Albert Hall concert and a £200 legacy, so that the net decrease on the estimate for 1909 was only £276 compared with last year. These figures were very gratifying to him, because when the Committee of the King's Fund met, which it did very early in the month, he had to take upon himself the responsibility, on very imperfect figures, of estimating the result of the work of the League for the whole year. He had faith in the workers, and so said to himself, "Because it is a bad year they will not fail, and I shall tell the King's Fund we will make a grant of £19,000," and the returns proved this faith was justified. In South St. Pancras, which collected only £1 last year, thanks to the able leadership of Mr. and Mrs. George Alexander, they had done nobly, and raised £200. Other noteworthy increases were St. George's, Hanover Square, £60; Paddington, £121; Epsom and Esher, £93; Westminster, £196; Lewisham, £133. With regard to the decreases, Chelsea was one of the most comfortable residential centres in London, and he much hoped that next year many volunteers would come forward and place Chelsea high on the list. He wished to remind his hearers that during the ten years just ended the League had given to the King's Fund a sum of £135,000 for the London hospitals, and in addition it had made grants to the country hospitals of £5,476, the amount given this year to the latter hospitals being increased to nearly £2,000. So the League had distributed a grand total of £140,476 raised in small subscriptions. That he regarded as most gratifying. The League very much required the help of more workers, and he hoped one result of the report of that meeting would be that a number of men and women would come forward and send in their names to the hon. secretaries, or to himself at 29, Southampton Street, Strand, when, wherever they might reside, they would be introduced to the President or Lady President of the district to which they might belong. The year had been a difficult one, but he thought it could be claimed to have been a successful one, and it was so because of the spirit which had been put into the work by the officers and members of the League. He was sure that no one who might come forward and take up the work would repent having done so, but would, on the contrary, be glad, as many of those present were, that the League of Mercy had been instituted, and that they had been privileged to work in the cause of the sick. One great difficulty was caused by old workers resigning; and he felt that if he could get into personal touch with them they would come back to the work. He (Sir Henry) thought all the workers should be personally seen, and encouraged to go on doing their best for the work to the end of their lives. More money was wanted—everybody wanted more money—(laughter)—and he would call the attention of Londoners to the probability that there was more vitality in the out-districts and a greater tendency to increase than in the Metropolis itself. He rejoiced to know that Lady Dimsdale was now working so well in the City that other districts must look to their laurels, or they would find the City would take first place. He hoped that would not be regarded as a discouragement, but rather as an inspiration to go forward. So far as his colleagues at the central office and he himself were concerned, they were prepared to throw themselves heartily into the work, and to encourage an output of energy in the Metropolitan districts by sending speakers or visiting the districts themselves, and thus do everything possible to make the year 1910 better than any of its predecessors. (Applause.)

Sir William Collins M.P., as one of the Honorary Secretaries, said there were a few facts in regard to the work which had not yet been mentioned. Though the King's Fund Committee had deplored the falling off of subscriptions, he had hopes that even the £276 deficiency to the League might be made up by the end of the year, so that they might be able to say that in no one year had the League shown any falling off. Whatever might be the inimical causes at work to account for that falling off, and he was as ignorant of them as the Chairman himself—(laughter)—that cause did not appear to have operated upon the small subscriber. One of the Vice-Presidents took £20 16s. 9d. in 5,001 individual penny subscriptions. In another case, out of £185 3s. 7d., £98 9s. 1d. was in pence. Half the subscriptions this year came from Extra-Metropolitan districts. Though this year there had been no Albert Hall concert, yet the League had to thank friends for other efforts in the same direction. Reference had already been made to their Royal Highnesses' large garden party last July, when forty-eight ladies and gentlemen received the Order of Mercy, bringing the total number to 383. Among the special items were the children's fancy dress dance at the Hotel Cecil in January, realising £203.

A successful matinée at Garrick Theatre was organised by Lady Paget in January last, resulting in a profit of £137.

A Café Chantant organised by Mrs. Harold Hardy (a Lady Vice-President of the West Marylebone district) in February last, at which H.R.H. The Duchess of Albany was present, resulted in a profit of £75.

A matinée of "The Merry Widow," given by Mr. George Edwardes at Daly's Theatre in June, resulted in £163 being handed over to the funds of the League.

A fête in connection with the League at Reading, was held in July, at which H.H. Princess Victoria of Schleswig-Holstein was present, taking charge of one of the stalls.

An "Old English Fête," organised by Miss Brenda Girvin (a member of the Lewisham district) in July last, realised a profit of £140 9s.

A Garden Fête was held in August, by the kindness of Mr. R. W. Baxter (a Vice-President of the Brentford district), at Southall.

A successful Bazaar, organised by Mrs. Lamert (Hon. Secretary and Lady Vice-President of the Deptford-Brockley district) and opened by H.R.H. The Duchess of Albany (Lady President of the same district), was held on October 23, realising a profit of £112 13s. 10d.

A Grand Morning Concert, under the presidency of H.H. Princess Victoria of Schleswig-Holstein, in aid of the League, was held at Reading in November, at which Her Highness was present.

An Afternoon Concert was given in aid of the League, by the Duchess of Somerset (Lady President of Westminster), on December 9 last.

A Grand Hungarian Concert also took place, under the organisation of Mr. and Mrs. Louis Felberman (Vice-Presidents of Kensington-South and Brixton districts respectively), at the Galleries of the Royal Society of British Artists on December 10, resulting in a profit of over £40.

Arrangements have been made by ladies in several of the districts for Dances to be held at the Grafton Galleries on January 13 and February 7, 1910.

Dr. Henry G. Thompson, V.D., proposed, and Mr. Bertram S. Straus, M.P., seconded, the following resolution: "That the authorities of Westminster Hospital be thanked for their kindness in granting the use of the Board Room for this meeting," which was heartily carried.

Mr. Sidney Hoffnung-Goldsmid proposed, and Mr. Walter Reynolds seconded, the vote of thanks to the Chairman. The seconder urged a more systematic provision of amusements for the purpose of raising funds, and urged the formation of an Amusements Committee for that purpose. He declared his intention of personally trying by that means to wipe off Hampstead's deficit.

Sir Walter Johnson deprecated the carrying out of Mr. Reynolds's suggestion. In supporting the resolution he pointed out that Mr. Reynold's suggestion was not practicable, as it cut at the root of the principle of personal service which had made the League the success it was. An occasional concert or benefit performance as a very exceptional thing might be accepted with gratitude when offered, but to make the League a continuous success it must depend upon the personal service gladly rendered by a multitude of members who gave of themselves as well as of their substance in the days of health in the cause of the sick.

The resolution was carried.

The Chairman, in acknowledging the vote, agreed with Sir Walter Johnson's view, and thought there must be some limit to the degree to which the goodness and kindness of the theatrical and musical professions were taxed.

Mr. F. Poliskin kindly lent his private orchestra, which, under his leadership, played a selection of music before the meeting commenced, and afterwards whilst the company were at tea.

### GRANTS TO EXTRA-METROPOLITAN HOSPITALS.

With the sanction of H.R.H. The Prince of Wales (*Grand President of the League of Mercy*), the following grants have been made for 1909 to Extra-Metropolitan Hospitals by the League:—

Berkshire—Royal Berkshire Hospital, Reading, £200; Windsor and Eton Royal Dispensary, £25; Newbury District Hospital, £20; St. Luke's Home, Woodley, Reading, £10; Morrell Home, Wallingford, £5.

Buckinghamshire—Royal Bucks Hospital, Aylesbury, £30; Marlow Cottage Hospital, £5; Iver Langley and Denham Cottage Hospital, £5; Chalfont St. Peter's Cottage Hospital, £5; Chesham Cottage Hospital, £5.

Essex—Essex and Colchester General Hospital, £25;

Chelmsford Infirmary, £25; Romford Victoria Cottage Hospital, £10; Victoria Hospital, Southend-on-Sea, £10; Buckhurst Hill Medical and Surgical Home, £10; Braintree and Bocking Cottage Hospital, £5; Brentwood Cottage Hospital, £10; Brentwood Convalescent Home for Children, £5; Buckhurst Hill Village Hospital, £5; Eden Cottage Hospital, Hatfield Broad Oak, £5; Clacton-on-Sea Cottage Hospital, £5.

Hampshire—Royal South Hants Hospital, Southampton, £150; Royal Hants County Hospital, Winchester, £150; Royal Portsea, Portsmouth and Gosport Hospital, £75; Royal Victoria Hospital, Bournemouth, £20; Southampton Free Eye Hospital, £5; Royal Boscombe Hospital, Bournemouth, £20; Aldershot Hospital, £5; Fleet Cottage Hospital, £5.

Hertfordshire—Hertford County Hospital and General Infirmary, £50; West Herts Infirmary, Hemel Hempstead, £20; Queen Victoria Cottage Hospital, Welwyn, £5.

Kent—West Kent General Hospital, Maidstone, £30: Gravesend Hospital, £30: General Kent and Canterbury Hospital, £25: Tunbridge Wells General Hospital, £25; Folkestone Victoria Cottage Hospital, £15; Ashford Cottage Hospital, £10; Sevenoaks Hospital for Children with Hip Disease, £25; Beckenham Cottage Hospital, £10; Bromley Cottage Hospital, £10; Chislehurst and Cray Cottage Hospital, £10; General Hospital and Seamen's Infirmary, Ramsgate, £10; Kent County Ophthalmic Hospital, Maidstone, £10; Margate Victoria Home for Children, £5; Faversham Cottage Hospital, £5; Tunbridge Wells Homœopathic Hospital, £5; Tonbridge Cottage Hospital, £5; Bexley Cottage Hospital, £5; Erith, Crayford and Belevdere Cottage Hospital, £5; Livingstone Cottage Hospital, Dartford, £5; Sidcup Cottage Hospital, £5.

Middlesex—Enfield Cottage Hospital, £10; Hounslow Hospital, £10; Hanwell Cottage Hospital, £5; Hayes Cottage Hospital, £5 ; St. John's Hospital, Twickenham, £10; Teddington and Hampton Wick Cottage Hospital, £5.

Northamptonshire—Northampton General Hospital, £50.

Oxfordshire—Radcliffe Infirmary, Oxford, £150; Horton Infirmary, Banbury, £25; The Eye Hospital, Oxford, £10.

Suffolk—West Suffolk General Hospital, Bury St. Edmunds, £30; East Suffolk and Ipswich Hospital, £20; Lowestoft Hospital, £10; St. Leonard's Hospital, Sudbury, £10.

Surrey—Royal Surrey County Hospital, £75; Croydon General Hospital, £30; Cranleigh Village Hospital, £10; Egham Cottage Hospital, £10; Surbiton Cottage Hospital, £10; Woking, Victoria Cottage Hospital, £5; Caterham Cottage Hospital, £5; East and West Molesey Cottage Hospital, £5; Epsom and Ewell Cottage Hospital, £5; Kingston Victoria Hospital, £5; Leatherhead Victoria Memorial Cottage Hospital, £5; Purley Cottage Hospital, £5; Sutton Cottage Hospital, £5; Thames Ditton Cottage Hospital, £5; Carshalton District Hospital, £5; Trimmer's Cottage Hospital, Farnham, £5.

Sussex—Brighton, Sussex County Hospital, £75; Hastings, St. Leonards and East Sussex Hospital, £100; Royal Alexandra Hospital for Children, Brighton, £20; Haywards Heath Cottage Hospital, £10; Eastbourne, Princess Alice Memorial Hospital, £10; Lewes Dispensary and Infirmary, £10; Crawley Cottage Hospital, £5; Crowborough Cottage Hospital, £5; Horsham Cottage Hospital, £5; Brighton Ear and Throat Hospital, £5; Leaf Homœopathic Cottage Hospital, £5; Petworth Cottage Hospital, £5; Uckfield Cottage Hospital, £5—Total, £1,980.

